# Active surveillance and clinical analysis of anaphylaxis based on the China Hospital Pharmacovigilance System

**DOI:** 10.3389/fphar.2023.1180685

**Published:** 2023-07-11

**Authors:** Chengcheng Wang, Zejing Li, Yingying Yu, Maoyan Feng, Anchang Liu

**Affiliations:** ^1^ Department of Pharmacy, Qilu Hospital (Qingdao), Cheeloo College of Medicine, Shandong University, Qingdao, China; ^2^ Department of Otolaryngology Head and Neck Surgery, Qilu Hospital (Qingdao), Cheeloo College of Medicine, Shandong University, Qingdao, China

**Keywords:** active surveillance, anaphylaxis, China Hospital Pharmacovigilance System, epinephrine, Delphi method

## Abstract

**Objective:** This study aimed to develop active surveillance programs (ASPs) for anaphylaxis using the China Hospital Pharmacovigilance System (CHPS) and analyze the characteristics, allergens, and management strategies for anaphylaxis within a tertiary hospital setting in China.

**Methods:** We retrospectively analyzed the anaphylaxis cases reported to the National Adverse Drug Reaction Monitoring System in our hospital from 2014 to 2021. Characteristic medical orders, progress notes, and diagnoses in these cases were recorded to identify initial anaphylaxis trigger entries. Based on these initial entries, the questionnaire was developed, and the Delphi method was used to establish consensus entries for anaphylaxis triggers. The CHPS was used to program these trigger entries and construct ASPs, which were then tested on the 238,194 discharged patients to evaluate their performance and analyze the related clinical data.

**Results:** Ten anaphylaxis triggers and three ASPs were ultimately identified. The ASPs captured 309 cases, out of which 94 cases were confirmed as anaphylaxis following manual screening. After removing duplicates, we noted 76 patients who experienced anaphylaxis 79 times. The positive rate of triggers and the positive predictive value of the programs were 0.13% and 30.42%, respectively. The incidence of anaphylaxis in our study was 0.03%, and the number of anaphylaxis cases detected by the ASPs was 5.64 times higher than those detected by the spontaneous reporting system. Anaphylaxis was more common among female patients. Antibacterial drugs, antineoplastic drugs, and contrast media were the most prevalent allergens in clinical practice. Anaphylaxis to antineoplastic drugs had the highest incidence (0.6%) when compared with patients admitted during the same period. Our study revealed a significant underuse of epinephrine and overuse of second-line therapy (glucocorticoids and antihistamines) in the management of anaphylaxis. Furthermore, we found the use and dosage of epinephrine to be inappropriate.

**Conclusion:** The CHPS can effectively utilize both structured and unstructured data to construct anaphylaxis ASPs, and this could counteract the under-reporting by the spontaneous reporting system, the primary adverse reaction monitoring method in China. The treatment and management of anaphylaxis are currently inadequate and require improvement to reduce mortality risk.

## 1 Introduction

Anaphylaxis is a severe, potentially fatal, systemic allergic reaction that occurs suddenly after contact with an allergy-causing substance (Sampson et al., 2006), and it can lead to serious consequences if there is a delayed diagnosis and inappropriate treatments. Drugs are generally considered to be the main cause of anaphylaxis (Tejedor-Alonso MA et al., 2015), and despite its relative rarity as an adverse drug reaction (ADR), drug-induced anaphylaxis remains a leading cause of allergy-related deaths in adults (Lee and Vadas, 2011; Jerschow et al., 2014). In addition, with the introduction of new medications such as biologics, small-molecule drugs, and chemotherapeutic drugs, the incidence of hospitalization caused by drug-induced anaphylaxis continues to increase (Cardona et al., 2020; Muraro et al., 2022). During the past decade, there has been an advanced understanding of the diagnosis, pathogenesis, and treatment management of anaphylaxis (Dribin and Castells, 2022; Weiler et al., 2023), but significant data and knowledge gaps remain in key clinical care and research domains, such as population science, validated clinical or biomarker-based models that predict disease outcome, and acute management (Dribin and Castells, 2022; Dribin et al., 2022). These shortcomings are especially acute in China (Li et al., 2019), where there is a dearth of active surveillance studies and epidemiological data on anaphylaxis. Additionally, studies showed gaps in the initial treatment of anaphylaxis between China and international guidelines (Jiang et al., 2020).

**FIGURE 1:**
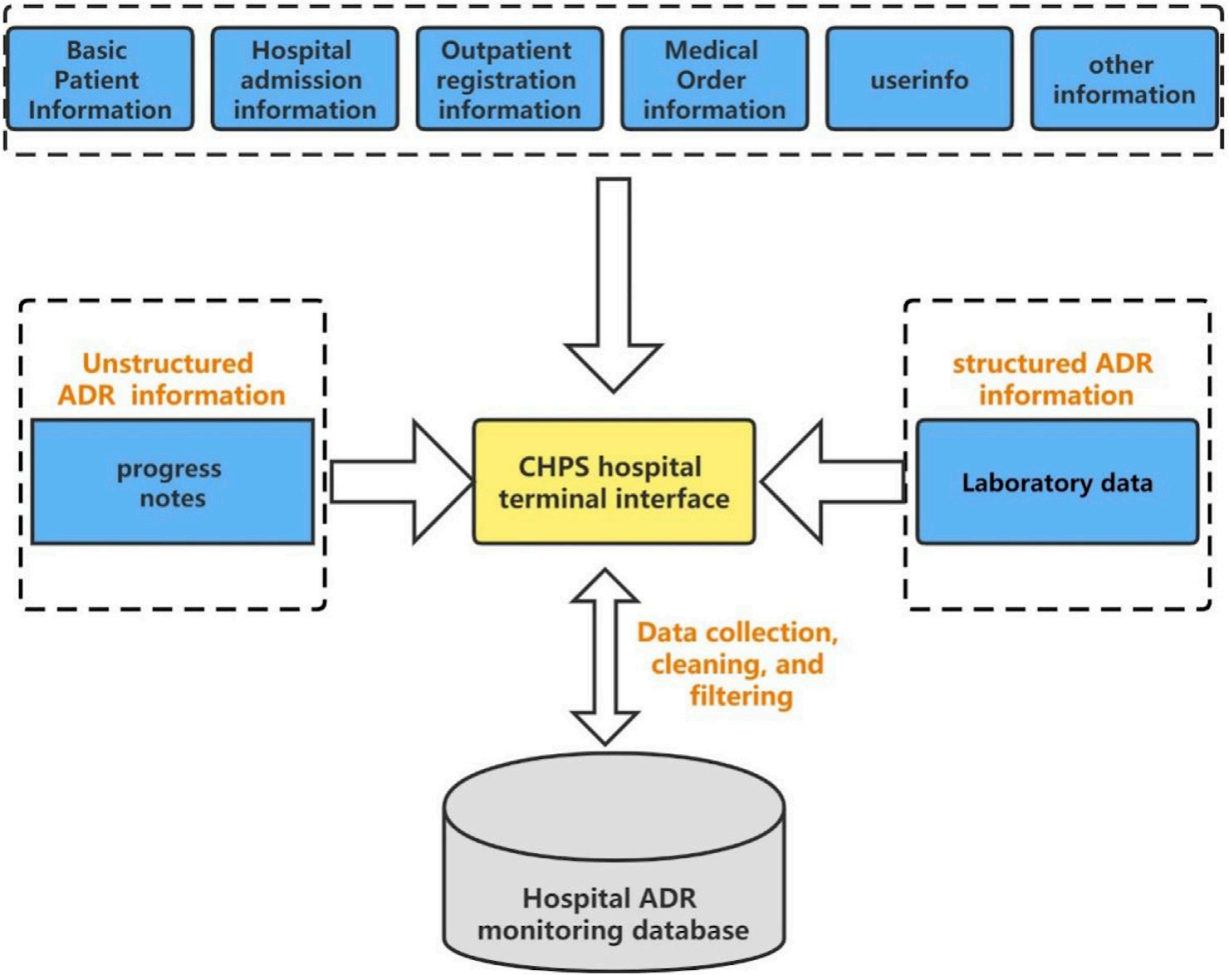
Data acquisition structure diagram of the CHPS.

The China Hospital Pharmacovigilance System (CHPS), launched and promoted by the China National Center for ADR Monitoring since 2016, possesses the capability to automatically collect and analyze data extracted from electronic hospital information systems (HISs) in sentinel hospitals (Figure 1) (Li et al., 2018). These data include a myriad of information, spanning diagnoses, medical orders, progress notes, test and examination results, and other information. The connection to the HIS makes it possible to simply, actively, and comprehensively obtain real-world drug safety data. At present, the CHPS encompasses more than 400 hospitals across China and is utilized in drug safety research owing to its high operability and accessibility (Li et al., 2018; Sun et al., 2020). Sun et al. (2020) utilized the CHPS to conduct a retrospective analysis of ADRs among 217 COVID-19 patients in China. The study underscored the CHPS’s critical role in actively monitoring and detecting ADR signals that reflect real-world ADRs during COVID-19 treatment, thereby providing valuable insights for ensuring safe medication in clinical settings.

This study aims to develop anaphylaxis triggers using the Delphi method and construct ASPs based on the CHPS. These ASPs are then applied to a cohort of 238,194 discharged patients, thereby facilitating an evaluation of their effectiveness and a detailed analysis of anaphylaxis characteristics, implicated allergens, and associated management practices within the Chinese population.

## 2 Materials and methods

### 2.1 Retrospective analysis of ADR reports

ADR reports from our hospital, spanning from January 2014 to December 2021, were retrieved from the National Adverse Drug Reaction Monitoring System. These reports were then retrospectively reviewed by both a pharmacist (CCW) and an allergist (ZJL). In reference to the diagnostic criteria for anaphylaxis (Sampson et al., 2006) (Table 1) and the Technical Specifications and Evaluation Criteria for Common Serious Adverse Drug Reactions issued by the National Center for ADR, China, the reviewers identified anaphylaxis cases and filled in the electronic case report forms. These forms included various details, such as diagnoses, departments, characteristic medical orders, and progress notes. After analyzing the relevant data, we formulated initial trigger entries for anaphylaxis.

**TABLE 1:** Diagnostic criteria for anaphylaxis.

Anaphylaxis is highly likely when any one of the following three criteria is fulfilled
1. Acute onset of illness (minutes to several hours), with involvement of the skin, mucosal tissue, or both (e.g., generalized hives, pruritus or flushing, and swollen lips, tongue, or uvula)
AND AT LEAST ONE OF THE FOLLOWING: a. Respiratory compromise (e.g., dyspnea, wheeze-bronchospasm, stridor, reduced peak expiratory flow (PEF), and hypoxemia)
b. Reduced blood pressure (BP) or associated symptoms of end-organ dysfunction (e.g., hypotonia [collapse], syncope, and incontinence)
2. Two or more of the following that occur rapidly after exposure to a likely allergen for that patient (minutes to several hours): a. Involvement of the skin-mucosal tissue (e.g., generalized hives, itch-flush, and swollen lips, tongue, or uvula)
b. Respiratory compromise (e.g., dyspnea, wheeze-bronchospasm, stridor, reduced PEF, and hypoxemia)
c. Reduced BP or associated symptoms (e.g., hypotonia [collapse], syncope, and incontinence)
d. Persistent gastrointestinal symptoms (e.g., crampy abdominal pain and vomiting)
3. Reduced BP after exposure to a known allergen for that patient (minutes to several hours): a. Infants and children: low systolic BP (age specific) or greater than 30% decrease in systolic BP
b. Adults: systolic BP of less than 90 mm Hg or greater than 30% decrease from that person’s baseline

### 2.2 Designing the expert consultation questionnaire

An expert consultation questionnaire was conceived, taking into account the initial entries. The questionnaire was structured in two sections; the first collected fundamental information about the experts, encompassing their field of specialization, educational background, years of professional experience, and academic title. The second section sought expert evaluation on the importance, familiarity, and judgment basis of trigger entries. A multidisciplinary panel of experts, each representing the fields of allergology, dermatology, emergency medicine, cardiology, intensive care, respiratory medicine, neurology, and pharmacy, was assembled. All panelists were selected based on their extensive experience in the diagnosis and treatment of anaphylaxis.

### 2.3 Employing the Delphi method for trigger entries

All experts were asked to rate the importance and familiarity of each item on a 5-point Likert scale (with 1 meaning strongly disagree, 2 meaning agree, 3 meaning neutral, 4 meaning agree, and 5 indicating strongly agree). The basis of judgment was classified into four categories: theoretical analysis, practice, informed by domestic and foreign peers, and intuition. A gradation from 0.1 to 0.5 points was allocated in accordance with the degree of impact on expert judgment, with the highest score of 0.5 being awarded when practice considerably influenced expert opinion. Furthermore, panel members were encouraged to submit free-text comments to clarify their responses to every question, suggest additional questions, or recommend modifications to the existing queries. The indicators of the Delphi method include the experts’ positive coefficient, the degree of expert authority, the concentration of expert opinions, and the degree coordination among expert opinions (Huan-huana et al., 2017). The positivity coefficient of experts was represented as the recovery rate of the questionnaire. The authority coefficient of experts, denoted as Cr, was dictated by the judgment basis of the entries (Ca) and the degree of familiarity with the consultation content (Cs), wherein Cr was given by the equation $Cr=\left(Ca+Cs\right)/2$ , and Cr values of 0.7 or higher were generally considered to carry a high degree of credibility. The concentration of expert opinion was depicted by the mean value of the importance score (Mj) and full score frequency (Kj) of the trigger entries. The cut-off value of Mj and Kj = mean-standard deviation, and those with scores higher than the cut-off value were included. The degree of expert opinion coordination was expressed as the coefficient of variation (Vj). The cut-off values of Vj = mean + standard deviation and those with scores lower than the cut-off value were included. Entries that failed to satisfy any of the three criteria were subsequently eliminated (Zeng, 1996).

### 2.4 Construction of ASPs

**FIGURE 2:**
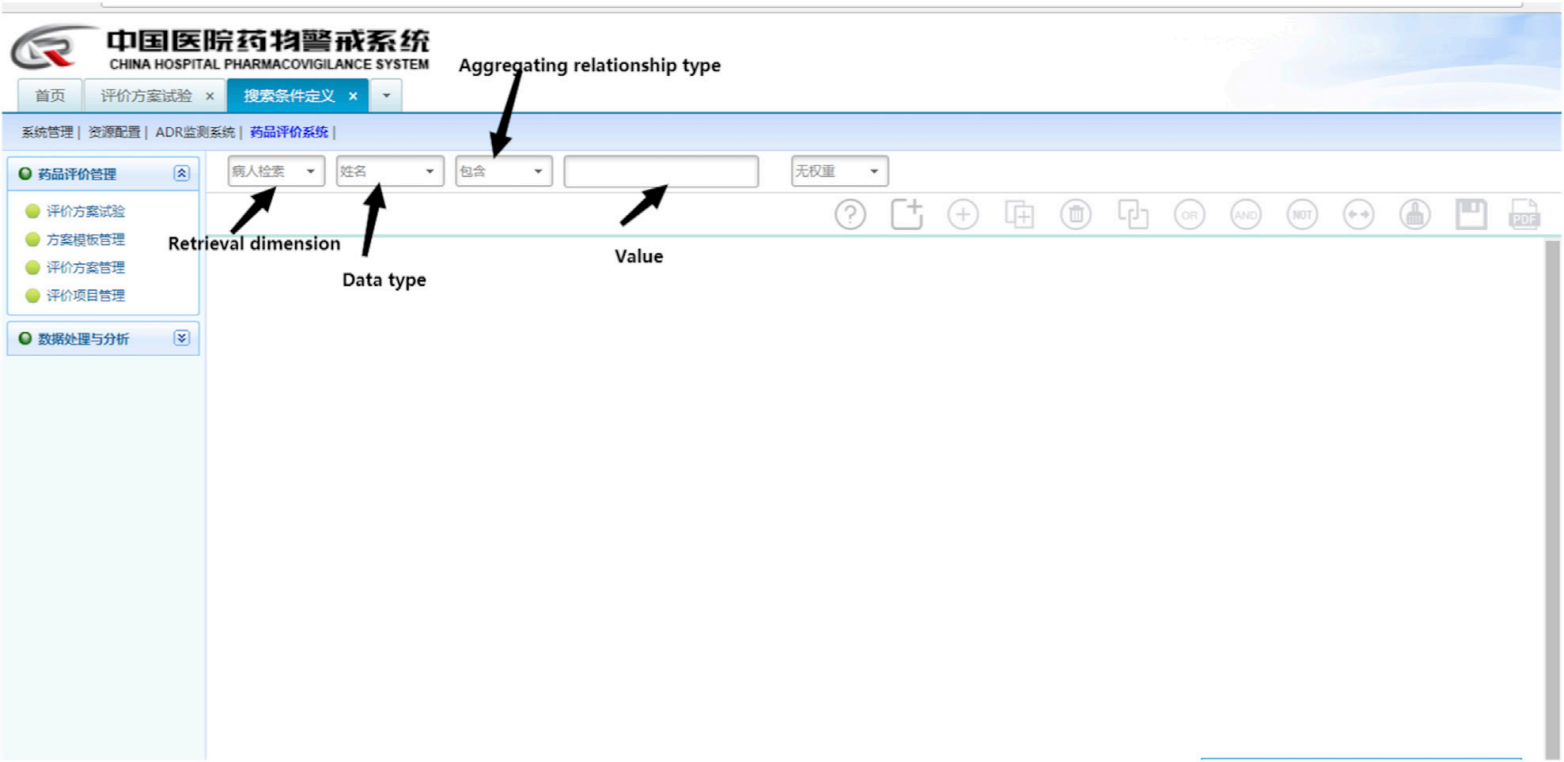
Website of the CHPS drug evaluation system.

The CHPS Drug Evaluation System (Figure 2), a subsystem of the CHPS, procures seven dimensions of clinical data from the HIS. These dimensions include patient information retrieval (basic information about patients), test retrieval (test items and test values), medical order retrieval (drug ID), medical record retrieval (admission records and progress notes), diagnosis retrieval, physical sign retrieval, and examination retrieval, and these seven dimensions can be easily connected with each other by Boolean logic operators. In this study, we utilized Boolean logic programming to formulate retrieval rules for triggers within medical orders, diagnoses, and progress notes. To augment the positive rate, triggers embedded in the progress notes and medical orders were conjoined by an “AND”operator.

**FIGURE 3:**
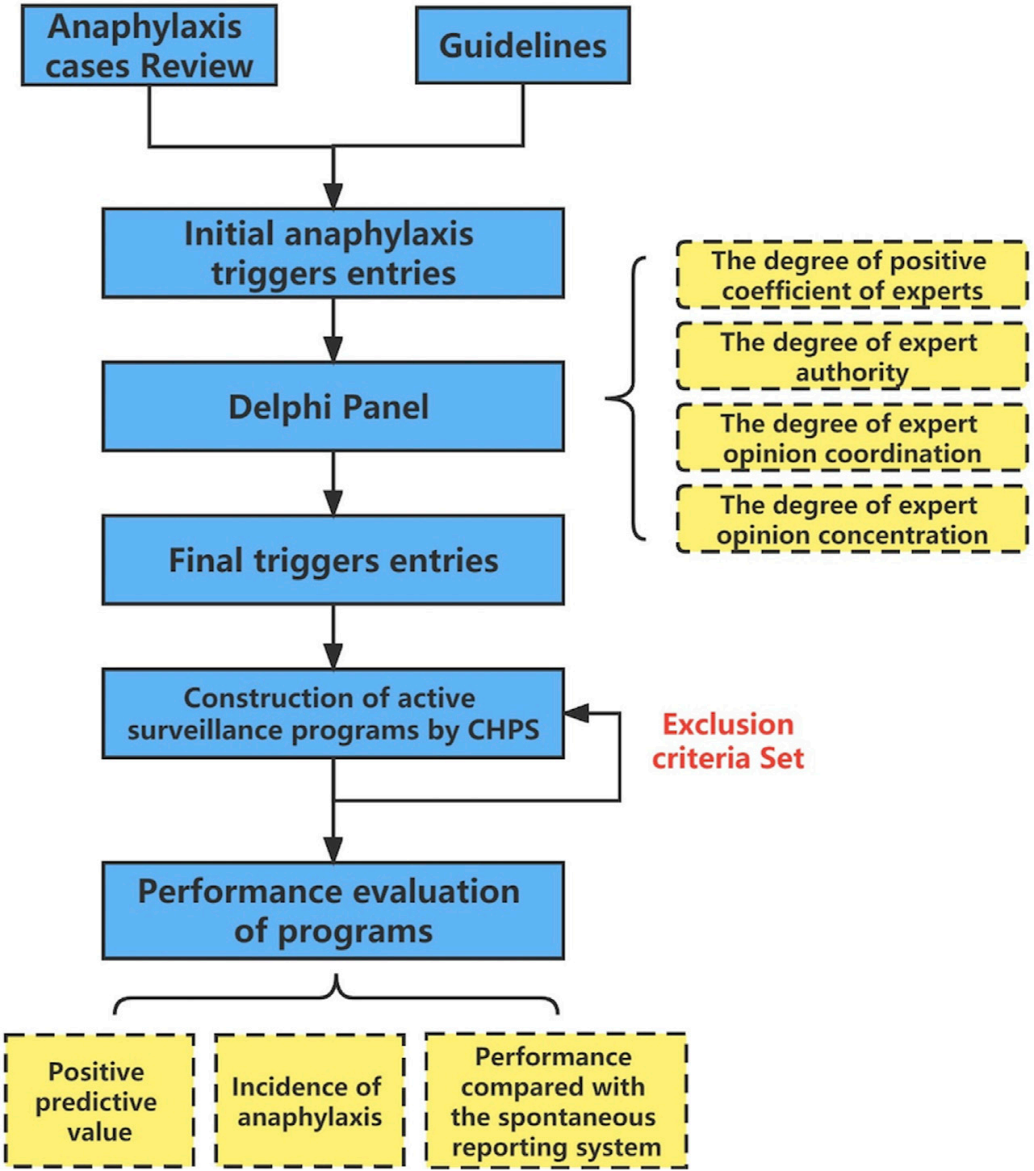
Flow chart of active surveillance for anaphylaxis.

These retrieval rules were then applied to discharged patients to obtain trigger-positive patient cases. Two reviewers, a pharmacist (CCW) and an allergist (ZJL), independently examined the results of the automated screening and jointly decided whether the cases were anaphylaxis. Cases were categorized as false positive if both reviewers considered them to be non-anaphylaxis. In the event of disagreement, a third more sophisticated reviewer with more experience (MYF) was consulted to make the final decision. Thereafter, the cases of false positives were analyzed, and exclusion rules were established to enhance the performance of the triggers. Ultimately, ASPs were constructed by integrating retrieval rules and exclusion rules (Figure 3).

### 2.5 Performance evaluation of ASPs

Upon running the ASPs, we manually reviewed the positive-triggered cases one by one, consequently establishing a comprehensive database for patients suffering from anaphylaxis. Furthermore, we calculated the count of anaphylaxis cases identified by ASPs to assess the performance of our system. The positive predictive value (PPV) of the ASPs was computed by dividing the number of anaphylaxis cases identified by the ASPs by the number of cases with positive triggers. The incidence of anaphylaxis was determined by dividing the number of anaphylaxis cases identified by ASPs by the total number of discharged patients. To quantify the efficiency of the ASPs relative to the spontaneous reporting system, we devised a ratio of the anaphylaxis cases detected by the ASPs to the anaphylaxis reports lodged within the spontaneous reporting system over an identical time frame.

### 2.6 Analysis of clinical data

We created additional electronic case report forms to extract various patient information, including patient ID, gender, age, the department of hospitalization, the time of anaphylaxis occurrence, descriptions of anaphylaxis processes in progress notes, suspected drugs, and the types and usage of therapeutic drugs. Furthermore, the suspected drugs were classified according to their pharmacological effects, and patients in the anaphylaxis database were categorized based on whether they experienced anaphylactic shock.

### 2.7 Statistic analysis

Categorical data were described by frequency counts and percentages. Continuous variables were depicted as means with standard deviation. Numerical differences between groups were assessed by the Chi-square test for categorical variables. The threshold for statistical significance was defined as *p* < 0.05. All statistical analyses were conducted using SPSS, Version 25.0 (SPSS Inc., Chicago, IL, United States)

## 3 Results

### 3.1 Trigger entries of anaphylaxis

From 2014 to 2021, our hospital reported 1827 ADR cases to the National Adverse Drug Reaction Monitoring System, and 22 cases were identified as anaphylaxis. We designed initial triggers using diagnoses, medical orders, and descriptions of progress notes. To refine trigger performance, we formulated exclusion rules. These included (1) the exclusion of descriptions of progress notes containing “anaphylactic shock” in informed consent prior to invasive procedures (such as anesthesia, bronchoscopy, and hematopoietic stem cells) and records aimed at preventing allergies, conducting allergy tests, and documenting allergy history; (2) the exclusion of the long-term medical order type for rescue drugs or when the interval between different rescue drug usage exceeded a day.

We distributed a 28-question online questionnaire to experts, and all eight questionnaires were effectively recovered, with a questionnaire recovery rate of 100%. Questionnaire data analysis yielded an expert authority coefficient of 0.92 ± 0.10, demonstrating high expert authority. The Mj, Kj, and Vj of the triggered entries are shown in Table 2. Finally, 10 trigger entries were developed by the Delphi method (Table 2).

**TABLE 2:** Trigger entries and scores for anaphylaxis.

Trigger entries	Mj (Cut-off = 3.90)	Kj (Cut-off = 0.36)	Vj (Cut-off = 0.37)
Diagnosis contains “anaphylactic shock”	5.00	1.00	0.00
Medical orders contain “epinephrine”	4.88	0.88	0.07
Medical orders contain “glucocorticoids (dexamethasone or methylprednisolone) combined with promethazine”	4.13	0.50	0.31
Progress notes contain “anaphylactic shock”	4.88	0.88	0.07
Progress notes contain “allergy”	4.38	0.63	0.17
Progress notes contain “cutaneous adverse reactions” (e.g., rash, flushing, pruritus)	4.38	0.63	0.20
Progress notes contain “respiratory system adverse reactions” (e.g., chest tightness, dyspnea, suffocation)	4.88	0.88	0.07
Progress notes contain “nervous system adverse reactions” (e.g., dizziness, irritability, unconsciousness, confusion)	3.80	0.50	0.63
Progress notes contain “digestive system adverse reactions” (e.g., nausea, vomiting, diarrhea)	3.50	0.13	0.22
Progress notes contain “circulatory system adverse reactions " (e.g., reduced BP, palpitation, precordial discomfort)	4.25	0.38	0.13

### 3.2 ASPs and performance

After using Boolean logic programming to integrate the trigger entries and optimizing the rules, the final surveillance programs were obtained (Table 3). The programs ran for approximately 3 min, automatically monitoring 238,194 medical records of discharge patients from 2018 to 2021, and 309 cases were positive for triggers, with a positive rate of 0.13%. After the manual screening, 94 cases of anaphylaxis were obtained, and the PPV was 30.42%. In total, 76 patients with 79 cases of anaphylaxis were finally obtained after removing the duplicates, including 37 cases of anaphylactic shock and 42 cases of no anaphylaxis shock. The incidence of anaphylaxis detected by the ASPs was 0.03%. During the same period, 14 cases of anaphylaxis were reported to the National Adverse Drug Reaction Monitoring System in our hospital. The number of anaphylaxis cases detected by the ASPs was 5.64 times higher than that detected by the spontaneous reporting system, and the under-reporting rate of the spontaneous reporting system was 83.72%.

**TABLE 3:** Active surveillance programs for anaphylaxis and its performance.

Items in surveillance programs	Positive frequency of triggers	Frequency of anaphylaxis	PPV (%)
Diagnosis contains “anaphylactic shock”	31	30	96.78
Progress notes contain “anaphylactic shock" and medical orders contain “epinephrine or glucocorticoids combined with promethazine”	19	11	57.89
Progress notes contain “allergy" and “adverse skin or respiratory or nervous system or digestive or circulatory system reactions,” and medical orders contain “epinephrine or glucocorticoids combined with promethazine”	259	53	20.46
Total	309	94	30.42

### 3.3 Characteristics of anaphylaxis

Among the detected cases (Table 4), 25 (31.65%) were males and 54 (68.35%) females, with a mean age of 55.78 years (range: 4–79 years). The highest incidence of anaphylaxis occurred in the emergency department (19 cases, 24.05%), succeeded by the oncology (12 cases, 15.19%) and gynecology departments (8 cases, 10.13%). It should be noted that all anaphylaxis in the gynecology department was caused by antineoplastic drugs.

**TABLE 4:** Demographic characteristics of anaphylaxis.

Demographic characteristics	Numbers
**Age**	55.78 ± 17.56
**Gender**	
Male	25 (31.65%)
Female	54 (68.35%)
**Department(n ≥ 2**)	
Emergency department	19 (24.05%)
Oncology department	12 (15.19%)
Gynecology department	8 (10.13%)
Cardiology department	5 (6.33%)
Neurology department	4 (5.06%)
Hepatology department	4 (5.06%)
Critical care medicine	3 (3.80%)
Neurosurgery department	3 (3.80%)
Respiratory department	3 (3.80%)
Hematology department	2 (2.53%)
Gastroenterology department	2 (2.53%)
Bone tumor department	2 (2.53%)
Pediatrics department	2 (2.53%)
Obstetrics department	2 (2.53%)
Anorectal department	2 (2.53%)

### 3.4 Anaphylaxis allergens

Out of the 79 anaphylaxis cases, 66 were drug-induced, constituting 83.54% of all anaphylaxis cases (Table 5). Others included eight cases with unidentified allergens, three cases were animal-induced (insect and shrimp allergens), and two cases were caused by absolute alcohol and irritating odors. Antibacterial drugs were the most common class of allergenic drugs, with cephalosporins being the most frequent, followed by antineoplastic drugs and contrast media. The drug with the highest individual frequency identified by the ASPs was oxaliplatin (5 cases, 7.58%), followed by carboplatin (4 cases, 6.06%). Additionally, traditional Chinese medicine injections, a class of drugs under special management in China due to safety concerns, were also commonly associated with anaphylaxis.

**TABLE 5:** Drugs that induced anaphylaxis.

Drug classification	Drug	Number
Antibacterial drugs	11 cephalosporins (four unspecified cephalosporins, three cefoperazone–sulbactam, three cefotiam, and one cefuroxime), three piperacillin–tazobactam, two amoxicillin, one metronidazole, and one levofloxacin	18
Antineoplastic drugs	Five oxaliplatin, four carboplatin, three doxorubicin liposome, two cetuximab, one nedaplatin, and one infliximab	16
Contrast media	Three iodixanol, three iopromide, and one iodine contrast agent with unknown details	7
Traditional Chinese medicine injections	Two Shenmai injections and one Xingnaojing injection	3
Glucocorticoid	Two dexamethasone and one methylprednisolone	3
Blood products	Two plasma and one platelet	3
Other drugs	One potassium sodium dehydroandroandrographolide succinate for injection, one extract of *Ginkgo biloba* leaf injection and citicoline, one reduced glutathione, one combined with compound paracetamol and amantadine hydrochloride, Qingre Sanjie capsule, Ganmao Qingre granule, one muscle relaxant, one lansoprazole, one epinastine and pantoprazole, one lidocaine, one domperidone, one iron sucrose, two transcatheter artery chemoembolization related drugs, one radionuclide, and one Zhenggu Zijin Wan	14
Unclear drugs	2	2
Total		66

### 3.5 Treatment regimen

Glucocorticoids (66 cases, 83.54%) were the most commonly used therapeutic drugs for patients with anaphylaxis, followed by promethazine (46 cases, 58.23%) and epinephrine (35 cases, 44.30%). Other drugs, including vitamin C injection (18 cases, 22.78%) and calcium gluconate (16 cases, 20.25%), were also utilized in the treatment of anaphylaxis (Table 6).

**TABLE 6:** Drugs for the treatment of anaphylaxis.

Treatment drugs	Case numbers (%)
Glucocorticoids (dexamethasone, methylprednisolone, and betamethasone	66 (83.54%)
Promethazine	46 (58.23%)
Epinephrine	35 (44.30%)
Vitamin C	18 (22.78%)
Calcium gluconate	16 (20.25%)
Others (dopamine, norepinephrine, etc.)	17 (21.52%)

A total of 35 anaphylaxis cases treated with epinephrine were analyzed, and there was a statistically significant difference in the epinephrine usage rate between patients with anaphylactic shock and those with non-anaphylactic shock (*p* < 0.01) (Table 7). The main route of epinephrine administration was intramuscular injection (45.71%), with subcutaneous injection (28.57%), bolus (25.71%), and other routes, but the epinephrine dose varied widely (Table 8).

**TABLE 7:** Epinephrine use in patients with anaphylaxis.

Patient classification	With epinephrine	Without epinephrine	*p*-value
Anaphylactic shock	29	8	<0.01
Non-anaphylactic shock	6	36	

**TABLE 8:** Dosage and administration of epinephrine.

Dosage and administration of epinephrine		Number (%)
Intramuscular injection	0.5 mg	10 (28.57%)
	0.3 mg	3 (8.57%)
	4 mg	1 (2.86%)
	1 mg	1 (2.86%)
	0.4 mg	1 (2.86%)
Subcutaneous injection	0.5 mg	4 (11.43%)
	0.3 mg	3 (8.57%)
	1 mg	2 (5.71%)
	0.15 mg	1 (2.86%)
Bolus	1 mg	2 (5.71%)
	0.02 mg	2 (5.71%)
	0.25 mg	1 (2.86%)
	0.2 mg	1 (2.86%)
	0.1 mg	1 (2.86%)
	0.03 mg	1 (2.86%)
	Unknown	1 (2.86%)

## 4 Discussion

Anaphylaxis constitutes an acute, potentially fatal systemic allergic reaction. Measuring and evaluating epidemiological data related to anaphylaxis is an important way to identify disease burden trends and risk factors. At present, epidemiological data sources for anaphylaxis encompass the purchase of epinephrine auto-injectors, national databases, primary care databases, representative sample surveys from the general population, hospital admissions, and emergency department visits (Tejedor Alonso et al., 2015; Tejedor-Alonso MA et al., 2015; Tanno et al., 2018), and hospital admission datasets were deemed the largest and most robust data available to understand trends in anaphylaxis (Turner et al., 2020). Research grounded on hospitalizations typically employs structured data such as the International Classification of Diseases (ICD)-9 and ICD-10 to identify patients with anaphylaxis (Mulla et al., 2011). Nonetheless, such studies are prone to drawbacks like misdiagnosis and misclassification (Tanno et al., 2018), which subsequently lead to an underestimation of anaphylaxis incidence. For example, Klein and Yocum (1995) undertook a retrospective analysis of patient records from the emergency department, uncovering 17 cases of anaphylaxis. However, only four of these 17 patients received an anaphylaxis diagnosis identifiable by ICD-9.

In this study, we devised trigger entries for anaphylaxis encompassing both structured (e.g., medical orders and diagnostic data) and unstructured (e.g., progress notes). This incorporation of unstructured data led to a two-fold rise in the anaphylaxis detection rate compared to the reliance solely on diagnosis-based structured data, thereby substantially augmenting the performance of the programs. Concurrently, when compared with the spontaneous reporting system of our hospital during the same period, it was evident that 83.72% of anaphylaxis cases were under-reported. This finding underscores the significant potential of ASPs to rectify the deficiencies of the spontaneous reporting system, the primary monitoring method for adverse reactions in China. The study by Panesar et al. (2013) illustrated that the incidence rates for anaphylaxis in Europe fluctuated between 1.5 and 7.9 per 100,000 person-years. Our research found the incidence of anaphylaxis in the Chinese population to be 8.29 episodes per 100,000 person-years, a figure surpassing other studies (Bann et al., 2021; Nunes et al., 2022) reliant on electronic medical records, which signals the efficacy of the ASPs. However, the sensitivity of our programs remained suboptimal. We analyzed 4,874 medical records of discharged patients from our hospital from 1 December to 31 December 2020 and recorded all suspected ADRs (based on the progress notes and diagnoses). Out of these, three cases were identified as anaphylaxis, and only one case could be effectively tracked by the ASPs. Analysis of undetected anaphylaxis in the aforementioned discharged patients and the spontaneous reporting system (see Supplementary Table S1) revealed that all eight patients were not diagnosed with anaphylactic shock. Among these, five cases either received only dexamethasone treatment or did not receive any pharmacological intervention post-anaphylaxis. Furthermore, two cases lacked progress notes indicating an “allergy,” and one case, although marked as “allergy,” also had a “prevention” note, which accounts for their exclusion from ASP monitoring. Hence, it is crucial to standardize the management of anaphylaxis and medical record documentation to enhance the sensitivity of the detection method.

Regarding demographic characteristics, our study demonstrated that the incidence of anaphylaxis was significantly higher in females than in males. Taking into account the gender composition of patients during the same period, the ratio of male-to-female anaphylaxis incidence was 1:2.1. Banerji et al. (2014) reported a similar gender disparity, with 71% of 716 anaphylaxis patients being female. Studies have indicated that anaphylaxis in females is less frequent than in males before puberty but increases rapidly and surpasses male incidence with age, although the exact mechanism is yet to be deciphered (Simons et al., 2002; Sheikh et al., 2008).

Death rates from drug-induced anaphylaxis have risen 300% over the last decade (Tejedor Alonso et al., 2015), and drugs associated with anaphylaxis vary based on different populations, time, geographic regions, drug usage patterns, genetic factors, anaphylaxis definitions, case registries, and study designs (Giavina-Bianchi et al., 2018). In our study, drugs were responsible for a significant 83.54% of all anaphylaxis cases, and the leading drug classes linked with anaphylaxis were antibacterial drugs, antineoplastic drugs, and contrast media. When compared with the number of patients treated at our hospital during the same period, we observed that anaphylaxis triggered by antineoplastic drugs had the highest proportion (0.06%), trailed by antibacterial drugs (0.02%) and contrast media (0.02%). Among the antineoplastic drugs, oxaliplatin emerged as the most common trigger, a finding consistent with results from the Korean population (Park et al., 2017). Indeed, hypersensitivity reactions induced by oxaliplatin have garnered substantial attention (Aroldi et al., 2015; Otani et al., 2017; Rogers et al., 2019), leading the China National Medical Products Administration to revise the package insert in August 2021 (Administration and N.M.P, 2021). This revision included a black-box warning about potential severe allergic reactions, even death, associated with oxaliplatin. Antibacterial drugs, particularly beta-lactams, are recognized as the primary causes of anaphylaxis, with previous studies suggesting a lower incidence of anaphylaxis with cephalosporins than penicillins (Park et al., 2017; Giavina-Bianchi et al., 2018), and drugs containing amoxicillin have been reported as the most frequent anaphylaxis triggers to the FDA (Yu et al., 2021). However, our study observed that cephalosporins were the most frequently implicated drugs, likely due to prescription practices in our hospital. As routine skin tests are not advocated prior to the administration of cephalosporins, future research should focus on devising prediction methods for allergic reactions with heightened sensitivity and specificity.

Administering an immediate intramuscular injection of epinephrine into the mid-thigh area is the primary treatment strategy for anaphylaxis, regardless of the presence of shock, as outlined in multiple guidelines (Cardona et al., 2020; Muraro et al., 2022). For adults, the recommended dosage is 0.01 mg/kg of body weight, not exceeding a total dose of 0.5 mg. Importantly, subcutaneous injection is not recommended for emergency intervention because of its slower onset of action (Li et al., 2019). Furthermore, although glucocorticosteroids and antihistamines are frequently employed in managing anaphylaxis, they are only recommended as secondary treatment options per guidelines, and their routine usage remains a contentious issue. Current evidence suggests that glucocorticosteroids may not provide any benefit or might even be detrimental in the acute management of anaphylaxis (Cardona et al., 2020). In our study, we noted that the use of glucocorticosteroids and antihistamines significantly outpaced that of epinephrine in anaphylaxis management (83.54% vs. 44.30%, 58.23% vs. 44.30%, *p* < 0.01). Notably, the employment of epinephrine was significantly less common in non-shock cases compared to shock incidents. Moreover, the application and dosage of epinephrine were not rational, reflected by a high percentage of subcutaneous epinephrine injections and considerable dosage inconsistency. Jiang et al. (2020) similarly underscored the significant underutilization, inappropriate usage, and dosage of epinephrine and the unreasonably high employment of glucocorticoids in China. Hence, it is crucial to improve anaphylaxis management and treatment by medical professionals to reduce mortality from this severe allergic reaction.

Our study does possess several limitations. Primarily, as a single-center study, the formulation of triggers in medical orders was based on the prescribing habits of doctors in our hospital; this context-specific design may compromise its external validity. Thus, when attempting to apply these triggers to other hospitals, certain elements may require modification. Additionally, our ASPs may not have captured all anaphylaxis cases due to certain inherent limitations, which could potentially affect the thoroughness of our results. This factor may have subtly influenced the outcomes of our research.

## Data Availability

The original contributions presented in the study are included in the article/Supplementary Material; further inquiries can be directed to the corresponding author.
