# Supplementary material for: Active surveillance and clinical analysis of anaphylaxis based on the China Hospital Pharmacovigilance System
Source: Front Pharmacol. 2023 Jul 11;14:1180685. doi: 10.3389/fphar.2023.1180685 (PMC10366353; doi:10.3389/fphar.2023.1180685)
Supplement: Supplementary file 1 [file Table1.DOCX]

Supplementary Material

Active Surveillance and Clinical Analysis of Anaphylaxis Based on China Hospital Pharmacovigilance System

Chengcheng Wang^1^, Zejing Li^2^, Yingying Yu ^1^, Maoyan Feng^1^, Anchang Liu^1*^

*** Correspondence:** Anchang Liu^*^acleu@126.com

# Supplementary Tables

**Table S1.** Anaphylaxis cases that did not detected by active surveillance programs

| No. | Drugs | **Department** | Reasons did not detected by active surveillance programs |
| --- | --- | --- | --- |
| 1 | cefotiam | Obstetrics department | Progress notes contain "precaution" |
| 2 | Vitamin K_1_ injection | Hepatology department | medical orders only contain glucocorticoids |
| 3 | Iohexol Injection | Endocrinology department | medical orders only contain glucocorticoids |
| 4 | Xingnaojing injection | Neurosurgery department | medical orders only contain glucocorticoids |
| 5 | saxagliptin | Respiratory department | Progress notes did not contain "allergy" |
| 6 | Compound Codeine Phosphate and Ibuprofen Sustained Release Tablets | Orthopedics department | Progress notes did not contain "allergy" |
| 7 | Ligustrazine Hydrochloride for injecetion | Rheumatology department | medical orders contain neither epinephrine nor glucocorticoids combined with promethazine |
| 8 | paclitaxel | General surgery department | medical orders contain neither epinephrine nor glucocorticoids combined with promethazine |
